# Supplementary figures and images for: Assessing perioperative risks in a mixed elderly surgical population using machine learning: A multi-objective symbolic regression approach to cardiorespiratory fitness derived from cardiopulmonary exercise testing
Source: PLOS Digit Health. 2025 May 16;4(5):e0000851. doi: 10.1371/journal.pdig.0000851 (PMC12084048; doi:10.1371/journal.pdig.0000851)

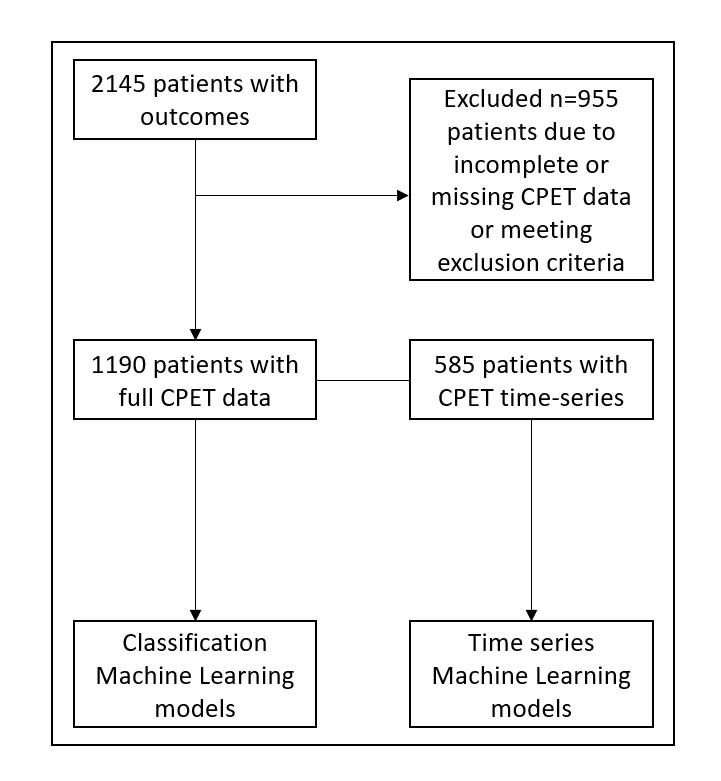

Supplement: S1 Fig — (TIF) [file pdig.0000851.s001.tif]

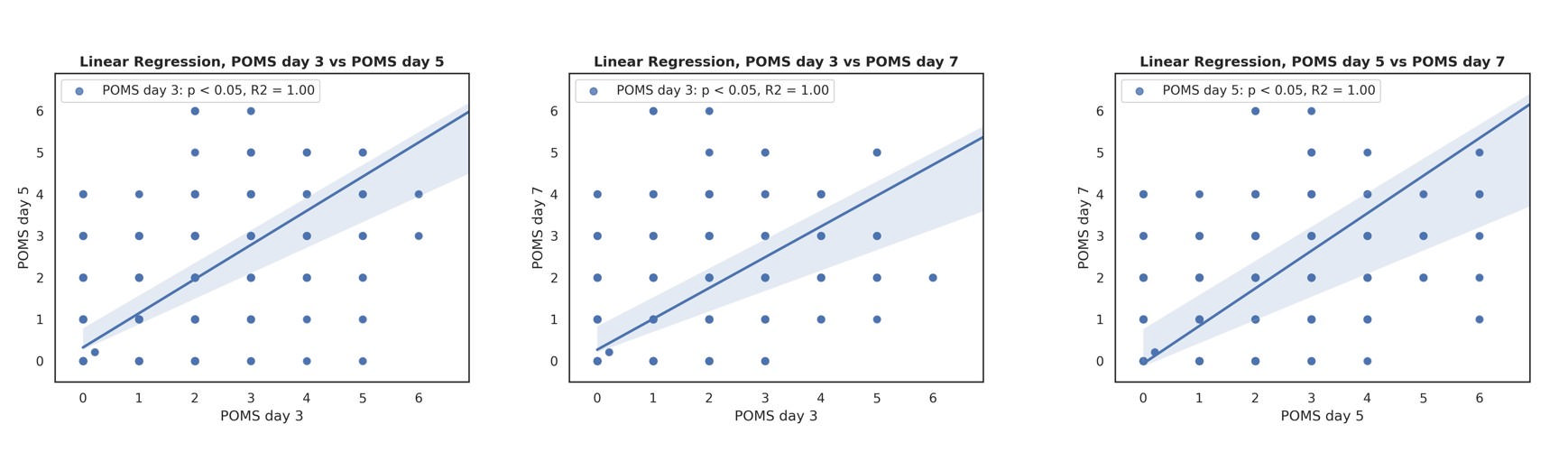

Supplement: S2 Fig — (TIF) [file pdig.0000851.s002.tif]

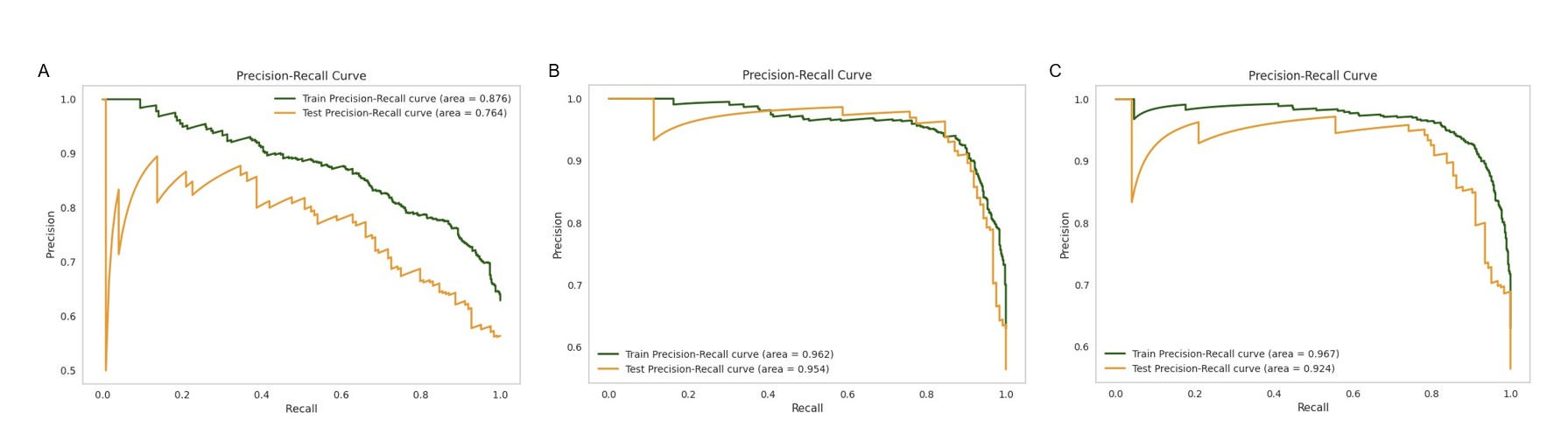

Supplement: S3 Fig — (TIF) [file pdig.0000851.s003.tif]
