# Supplementary material for: Assessing perioperative risks in a mixed elderly surgical population using machine learning: A multi-objective symbolic regression approach to cardiorespiratory fitness derived from cardiopulmonary exercise testing
Source: PLOS Digit Health. 2025 May 16;4(5):e0000851. doi: 10.1371/journal.pdig.0000851 (PMC12084048; doi:10.1371/journal.pdig.0000851)
Supplement: S1 Table — (DOCX) [file pdig.0000851.s004.docx]

**S1 Table:** Postoperative Morbidity Score (POMS)

| Morbidity type | Criteria | Source of data |
| --- | --- | --- |
| Pulmonary | The patient has developed a new requirement for oxygen or respiratory support. | Patient observation  Treatment chart |
| Infectious | Currently on antibiotics and/or has had a temperature of >38°C in the last 24 hr. | Treatment chart  Observation chart |
| Renal | Presence of oliguria <500 mL/24 hr; increased serum creatinine (>30% from preoperative level); urinary catheter in situ. | Fluid balance chart  Biochemistry result  Patient observation |
| Gastrointestinal | Unable to tolerate an enteral diet for any reason including nausea, vomiting, and abdominal distension (use of antiemetic). | Patient questioning  Fluid balance chart  Treatment chart |
| Cardiovascular | Diagnostic tests or therapy within the last 24 hr for any of the following: new myocardial infarction or ischemia, hypotension (requiring fluid therapy >200 mL/hr or pharmacological therapy), atrial or ventricular arrhythmias, cardiogenic pulmonary oedema, thrombotic event (requiring anticoagulation). | Treatment chart  Note review |
| Neurological | New focal neurological deficit, confusion, delirium, or coma. | Note review  Patient questioning |
| Hematological | Requirement for any of the following within the last 24 hr: packed erythrocytes, platelets, fresh-frozen plasma, or cryoprecipitate. | Treatment chart  Fluid balance chart |
| Wound | Wound dehiscence requiring surgical exploration or drainage of pus from the operation wound with or without isolation of organisms. | Note review  Pathology results |
| Pain | New postoperative pain significant enough to require parenteral opioids or regional analgesia. | Treatment chart  Patient questioning |
