# Supplementary material for: Assessing perioperative risks in a mixed elderly surgical population using machine learning: A multi-objective symbolic regression approach to cardiorespiratory fitness derived from cardiopulmonary exercise testing
Source: PLOS Digit Health. 2025 May 16;4(5):e0000851. doi: 10.1371/journal.pdig.0000851 (PMC12084048; doi:10.1371/journal.pdig.0000851)
Supplement: S2 Table — Comparison of Multi-Objective Symbolic Regression (MOSR), versus Decision Tree Classifier, Light Gradient Bosting Machine (LGBM) Classifier, Logistic Regression, Random Forest Classifier and Extreme Gradient Boosting (XGB) Classifier. Values are expressed in mean and 95% CI from 10 execution on training set. CRF: Cardiorespiratory Fitness, CI: 95% Confidence interval, PPV: Positive Predictive Value, NPV: Negative Predictive Value, ASA: American Society of Anaesthesiologist score, DASI: Duke Activity Status Index, CPET: Cardiopulmonary Exercise Testing, PPOSSUM: Portsmouth Physiological and Operative Severity Score for the enumeration of Mortality and morbidity. (DOCX) [file pdig.0000851.s005.docx]

**S2 Table: Machine learning model performances TRAINING set**

Comparison of Multi-Objective Symbolic Regression (MOSR), versus Decision Tree Classifier, Light Gradient Bosting Machine (LGBM) Classifier, Logistic Regression, Random Forest Classifier and Extreme Gradient Boosting (XGB) Classifier. Values are expressed in mean and 95% CI from 10 execution on training set. CRF: Cardiorespiratory Fitness, CI: 95% Confidence interval, PPV: Positive Predictive Value, NPV: Negative Predictive Value, ASA: American Society of Anaesthesiologist score, DASI: Duke Activity Status Index, CPET: Cardiopulmonary Exercise Testing, PPOSSUM: Portsmouth Physiological and Operative Severity Score for the enumeration of Mortality and morbidity.

| Model – CRF and Clinical Dataset | Accuracy | ±CI | AUC | ±CI | F1 Score | ±CI | Sensitivity | ±CI | Specificity | ±CI | PPV (precision) | ±CI | NPV | ±CI |
| --- | --- | --- | --- | --- | --- | --- | --- | --- | --- | --- | --- | --- | --- | --- |
| MOSR | 0.886 | ± 0.004 | 0.951 | ± 0.024 | 0.897 | ± 0.004 | 0.877 | ± 0.006 | 0.919 | ± 0.008 | 0.898 | ± 0.009 | 0.877 | ± 0.006 |
| Decision Tree Classifier | 1 | ± 0.0 | 1 | ± 0.0 | 1 | ± 0.0 | 1 | ± 0.0 | 1 | ± 0.0 | 1 | ± 0.0 | 1 | ± 0.0 |
| LGBM Classifier | 1 | ± 0.0 | 1 | ± 0.0 | 1 | ± 0.0 | 1 | ± 0.0 | 1 | ± 0.0 | 1 | ± 0.0 | 1 | ± 0.0 |
| Logistic Regression | 0.908 | ± 0.004 | 0.963 | ± 0.003 | 0.919 | ± 0.004 | 0.924 | ± 0.004 | 0.914 | ± 0.005 | 0.887 | ± 0.006 | 0.924 | ± 0.004 |
| Random Forest Classifier | 1 | ± 0.0 | 1 | ± 0.0 | 1 | ± 0.0 | 1 | ± 0.0 | 1 | ± 0.0 | 1 | ± 0.0 | 1 | ± 0.0 |
| XGB Classifier | 1 | ± 0.0002 | 1 | ± 0.0 | 1 | ± 0.0 | 1 | ± 0.0 | 1 | ± 0.0 | 1 | ± 0.0 | 1 | ± 0.0 |
|  |  |  |  |  |  |  |  |  |  |  |  |  |  |  |
| Model – CRF Dataset | **Accuracy** | **±CI** | **AUC** | **±CI** | **F1 Score** | **±CI** | **Sensitivity** | **±CI** | **Specificity** | **±CI** | **PPV (precision)** | **±CI** | **NPV** | **±CI** |
| MOSR | 0.888 | ± 0.003 | 0.943 | ± 0.002 | 0.901 | ± 0.002 | 0.883 | ± 0.008 | 0.918 | ± 0.005 | 0.895 | ± 0.004 | 0.883 | ± 0.036 |
| Decision Tree Classifier | 0.997 | ± 0.001 | 0.999 | ± 0.001 | 0.997 | ± 0.001 | 0.995 | ± 0.001 | 1 | ± 0.0 | 1 | ± 0.0 | 0.995 | ± 0.001 |
| LGBM Classifier | 0.997 | ± 0.001 | 0.999 | ± 0.001 | 0.997 | ± 0.001 | 1 | ± 0.0 | 1 | ± 0.0 | 0.998 | ± 0.001 | 1 | ± 0.0 |
| Logistic Regression | 0.883 | ± 0.001 | 0.946 | ± 0.003 | 0.897 | ± 0.005 | 0.905 | ± 0.004 | 0.890 | ± 0.007 | 0.854 | ± 0.010 | 0.905 | ± 0.004 |
| Random Forest Classifier | 0.997 | ± 0.001 | 0.999 | ± 0.001 | 0.997 | ± 0.001 | 0.995 | ± 0.005 | 1 | ± 0.0 | 1 | ± 0.0 | 0.995 | ± 0.001 |
| XGB Classifier | 0.997 | ± 0.001 | 0.999 | ± 0.001 | 0.997 | ± 0.001 | 0.995 | ± 0.001 | 1 | ± 0.0 | 1 | ± 0.0 | 0.995 | ± 0.001 |
|  |  |  |  |  |  |  |  |  |  |  |  |  |  |  |
| Model – Clinical Dataset | **Accuracy** | **±CI** | **AUC** | **±CI** | **F1 Score** | **±CI** | **Sensitivity** | **±CI** | **Specificity** | **±CI** | **PPV (precision)** | **±CI** | **NPV** | **±CI** |
| MOSR | 0.727 | ± 0.006 | 0.811 | ± 0.005 | 0.751 | ± 0.007 | 0.723 | ± 0.007 | 0.728 | ± 0.014 | 0.733 | ± 0.016 | 0.723 | ± 0.007 |
| Decision Tree Classifier | 1 | ± 0.0 | 1 | ± 0.0 | 1 | ± 0.0 | 1 | ± 0.0 | 1 | ± 0.0 | 1 | ± 0.0 | 1 | ± 0.0 |
| LGBM Classifier | 1 | ± 0.0 | 1 | ± 0.0 | 1 | ± 0.0 | 1 | ± 0.0 | 1 | ± 0.0 | 1 | ± 0.0 | 1 | ± 0.0 |
| Logistic Regression | 0.721 | ± 0.007 | 0.803 | ± 0.009 | 0.757 | ± 0.006 | 0.77 | ± 0.008 | 0.743 | ± 0.008 | 0.654 | ± 0.001 | 0.771 | ± 0.008 |
| Random Forest Classifier | 1 | ± 0.0 | 1 | ± 0.0 | 1 | ± 0.0 | 1 | ± 0.0 | 1 | ± 0.0 | 1 | ± 0.0 | 1 | ± 0.0 |
| XGB Classifier | 1 | ± 0.0 | 1 | ± 0.0 | 1 | ± 0.0 | 1 | ± 0.0 | 1 | ± 0.0 | 1 | ± 0.0 | 1 | ± 0.0 |
|  |  |  |  |  |  |  |  |  |  |  |  |  |  |  |
| Model – CRF-TS Dataset | **Accuracy** | **±CI** | **AUC** | **±CI** | **F1 Score** | **±CI** | **Sensitivity** | **±CI** | **Specificity** | **±CI** | **PPV (precision)** | **±CI** | **NPV** | **±CI** |
| MOSR | 0.783 | ± 0.056 | 0.869 | ± 0.0324 | 0.827 | ± 0.0375 | 0.787 | ± 0.0354 | 0.872 | ± 0.0387 | 0.775 | ± 0.124 | 0.787 | ± 0.0548 |
| Decision Tree Classifier | 0.998 | ± 0.001 | 0.999 | ± 0.004 | 0.998 | ± 0.001 | 0.997 | ± 0.001 | 1 | ± 0.0 | 1 | ± 0.0 | 0.997 | ± 0.001 |
| LGBM Classifier | 0.998 | ± 0.001 | 0.999 | ± 0.001 | 0.998 | ± 0.001 | 0.997 | ± 0.001 | 1 | ± 0.001 | 1 | ± 0.0 | 0.997 | ± 0.001 |
| Logistic Regression | 0.656 | ± 0.015 | 0.756 | ± 0.012 | 0.776 | ± 0.008 | 0.843 | ± 0.010 | 0.704 | ± 0.010 | 0.394 | ± 0.021 | 0.865 | ± 0.002 |
| Random Forest Classifier | 0.997 | ± 0.002 | 0.999 | ± 0.001 | 0.998 | ± 0.001 | 1 | ± 0.0 | 0.996 | ± 0.001 | 0.994 | ± 0.001 | 1 | ± 0.0 |
| XGB Classifier | 0.998 | ± 0.001 | 0.999 | ± 0.001 | 0.999 | ± 0.001 | 0.997 | ± 0.001 | 1 | ± 0.0 | 1 | ± 0.0 | 0.997 | ± 0.001 |
|  |  |  |  |  |  |  |  |  |  |  |  |  |  |  |
| Model – CRF 585 subset | **Accuracy** | **±CI** | **AUC** | **±CI** | **F1 Score** | **±CI** | **Sensitivity** | **±CI** | **Specificity** | **±CI** | **PPV (precision)** | **±CI** | **NPV** | **±CI** |
| MOSR | 0.685 | ± 0.0754 | 0.749 | ± 0.086 | 0.784 | ± 0.0583 | 0.914 | ± 0.067 | 0.686 | ± 0.0574 | 0.302 | ± 0.145 | 0.913 | ± 0.0831 |
| Decision Tree Classifier | 0.998 | ± 0.001 | 0.999 | ± 0.004 | 0.998 | ± 0.001 | 0.997 | ± 0.001 | 1 | ± 0.0 | 1 | ± 0.0 | 0.997 | ± 0.001 |
| LGBM Classifier | 0.998 | ± 0.001 | 0.999 | ± 0.001 | 0.998 | ± 0.001 | 0.997 | ± 0.001 | 1 | ± 0.001 | 1 | ± 0.0 | 0.997 | ± 0.001 |
| Logistic Regression | 0.688 | ± 0.012 | 0.712 | ± 0.011 | 0.776 | ± 0.008 | 0.865 | ± 0.010 | 0.704 | ± 0.010 | 0.394 | ± 0.021 | 0.865 | ± 0.010 |
| Random Forest Classifier | 0.997 | ± 0.002 | 0.999 | ± 0.001 | 0.998 | ± 0.001 | 1 | ± 0.0 | 0.996 | ± 0.001 | 0.994 | ± 0.001 | 1 | ± 0.0 |
| XGB Classifier | 0.998 | ± 0.001 | 0.999 | ± 0.001 | 0.999 | ± 0.001 | 0.997 | ± 0.001 | 1 | ± 0.0 | 1 | ± 0.0 | 0.997 | ± 0.001 |
