# Supplementary material for: Assessing perioperative risks in a mixed elderly surgical population using machine learning: A multi-objective symbolic regression approach to cardiorespiratory fitness derived from cardiopulmonary exercise testing
Source: PLOS Digit Health. 2025 May 16;4(5):e0000851. doi: 10.1371/journal.pdig.0000851 (PMC12084048; doi:10.1371/journal.pdig.0000851)
Supplement: Attachment — Submitted filename: Reviewer comments.docx [file pdig.0000851.s006.docx]

To the authors

Well done on an interesting and novel idea. As a perioperative physician I am always looking for ways to improve risk stratification through the combination of CPET, risk scores, models, biomarkers and now MOSR.

See comments and questions below.

| **General** |  |
| --- | --- |
| **Reviewer’s comments** | **Author’s comments** |
| Please review your references to ensure that they are complete, correct, standardised and in accordance with journal requirements. |  |
| Please use line numbering for next draft to aid comments/corrections. |  |

| **Title** |  |
| --- | --- |
| **Reviewer’s comments** | **Author’s comments** |
| Consider adding machine-learning into title as it is a more widely-used phrase that might catch peoples attention. Not many physicians may know what MOSR is yet. |  |

| **Key points** |  |
| --- | --- |
| **Reviewer’s comments** | **Author’s comments** |
| Meaning section: avoid saying “potentially improving patient outcomes”. The statement may be true but it is too broad and presumptuous. Rather say “and could improve peri-operative decision making” |  |

| **Introduction** |  |
| --- | --- |
| **Reviewer’s comments** | **Author’s comments** |
| Last sentence of discussion. You mention the word “predicting”. Rather use correlations or associations than predictions as that is more scientifically accurate. |  |

| **Methods** |  |
| --- | --- |
| **Reviewer’s comments** | **Author’s comments** |
| In some areas, you mention major surgery and other areas complex major surgery. Stick to one. |  |
| Patient selection, you say >18 years old but then the patients are elderly? |  |
| You switch between VO2 peak and peak VO2. Standardise throughout. |  |
| How was the quality of the data assessed? Was this independently or together? Was there a mediator for disagreements? |  |
| Why 80:20 first then 90:10? Only 10 test samples? |  |

| **Results** |  |
| --- | --- |
| **Reviewer’s comments** | **Author’s comments** |
| In comorbidities (table 1), include renal dysfunction if you have those numbers available because it is a commonly used parameter in many risk scores (Lee’s RCRI). |  |
| 69% of patients male. Why do you think such a big difference between males and females? |  |
| Mortality rates-Is this in keeping with other studies rates? As UCLH is a first-world tertiary hospital so care may be different to other hospitals which affects generalisability of findings (I know that this is mentioned as a limitation but maybe just comment on whether the mortality rate is similar to other hospitals?) |  |
| Table 2: some of the AUC values are very high? Makes me question the inputs. I think the model might need some re-working, especially overlapping inputs. |  |
| SHAP plot: it currently shows a lot of CPET dataset but needs to show more of the clinical dataset. |  |
| SHAP plot: why is work rate at AT and work rate at peak exercise inverse? Surely the higher the work rate at both times, the worse the outcomes? |  |
| SHAP plot: how was operation severity determined? |  |
| Figure 2F: why when clinical dataset combined with CRF dataset did it degrade model outcomes? |  |
| Figure 3A: was cleaning done on the dataset as outliers are clearly visible and could skew data. Also, this would better be shown as a box-whisker plot at each time interval. |  |
| Figure 3B: why was this smoothing done? Makes the picture prettier but might lose important nuances in dataset. |  |

| **Discussion** |  |
| --- | --- |
| **Reviewer’s comments** | **Author’s comments** |
| Cardiorespiratory fitness written out in full. Use CRF throughout. |  |
| Going forward, we want to use these models to counsel patients regarding their risk. A couple of questions/comments/potential research opportunities.   1) Can you perform surgery specific models vs general models to adequately counsel patients for individual surgeries 2) Consider incorporating biomarkers such as proBNP into future models |  |
