# Supplementary material for: Assessing perioperative risks in a mixed elderly surgical population using machine learning: A multi-objective symbolic regression approach to cardiorespiratory fitness derived from cardiopulmonary exercise testing
Source: PLOS Digit Health. 2025 May 16;4(5):e0000851. doi: 10.1371/journal.pdig.0000851 (PMC12084048; doi:10.1371/journal.pdig.0000851)
Supplement: Attachment — Submitted filename: Reviewer comments JW_EM_PA.docx [file pdig.0000851.s008.docx]

London, 18/03/25

Dear Leo Anthony Celi,

Editor-in-Chief, PLOS Digital Health,

I am pleased to re-submit our manuscript titled "Assessing Perioperative Risks in a Mixed Elderly Surgical Population Using Machine Learning: A Multi-Objective Symbolic Regression Approach to Cardiorespiratory Fitness Derived from Cardiopulmonary Exercise Testing" for consideration in PLOS Digital Health.

I would like to sincerely thank you for your insightful comments and valuable feedback. We have carefully considered each of your suggestions and made the necessary corrections as per your guidance.

Where applicable, we have addressed the changes in the manuscript and highlighted them in red for your convenience, with corresponding line numbers for clarity.

Your thoughtful input has greatly improved the quality of the paper, and we are truly grateful for your contributions.

Thank you once again for your time and effort.

Yours sincerely,

Dr. Pietro Arina

On behalf of the co-authors

To the authors

Well done on an interesting and novel idea. As a perioperative physician I am always looking for ways to improve risk stratification through the combination of CPET, risk scores, models, biomarkers and now MOSR.

See comments and questions below.

| **General** |  |
| --- | --- |
| **Reviewer’s comments** | **Author’s comments** |
| Please review your references to ensure that they are complete, correct, standardised and in accordance with journal requirements. | We have reviewed the references and ensured they are now complete, correct, and standardized according to the Vancouver style, as per the journal's requirements. |
| Please use line numbering for next draft to aid comments/corrections. | We have implemented line numbering in the next draft to facilitate comments and corrections, as requested. |

| **Title** |  |
| --- | --- |
| **Reviewer’s comments** | **Author’s comments** |
| Consider adding machine-learning into title as it is a more widely-used phrase that might catch peoples attention. Not many physicians may know what MOSR is yet. | Line 2: Assessing perioperative risks in a mixed elderly surgical population using machine learning: a multi-objective symbolic regression approach to cardiorespiratory fitness derived from cardiopulmonary exercise testing |

| **Key points** |  |
| --- | --- |
| **Reviewer’s comments** | **Author’s comments** |
| Meaning section: avoid saying “potentially improving patient outcomes”. The statement may be true but it is too broad and presumptuous. Rather say “and could improve peri-operative decision making” | The statement in the Meaning section (line 108) has been revised as requested. Instead of saying “potentially improving patient outcomes,” it now reads “and could improve peri-operative decision making” in line 113. |

| **Introduction** |  |
| --- | --- |
| **Reviewer’s comments** | **Author’s comments** |
| Last sentence of discussion. You mention the word “predicting”. Rather use correlations or associations than predictions as that is more scientifically accurate. | The last sentence of the discussion has been revised to replace the word "predicting" with "correlations" or "associations" for greater scientific accuracy. It now reads: " In our study the potential of MOSR for correlating patient clinical and physiological features with perioperative outcomes is highlighted, showcasing its capability to autonomously select important features and create models that are both simple and accurate, demonstrating strong generalizability" from line 466. |

| **Methods** |  |
| --- | --- |
| **Reviewer’s comments** | **Author’s comments** |
| In some areas, you mention major surgery and other areas complex major surgery. Stick to one. | The document has been revised to consistently use the term "major surgery" throughout, removing the word "complex" where it was previously used. |
| Patient selection, you say >18 years old but then the patients are elderly? | The patient inclusion criteria specify that individuals over 18 years old were included in the study. This criterion was set to exclude any potential patients under 18 years old from the main database. However, it is important to note that the study population is skewed towards the elderly since these are the predominant population undergoing major surgery in our institution |
| You switch between VO2 peak and peak VO2. Standardise throughout. | The document has been standardized to consistently use the term "VO2 peak" throughout. |
| How was the quality of the data assessed? Was this independently or together? Was there a mediator for disagreements? | In lines 155-157, we added: "The initial analysis was performed by two clinical exercise physiologists and was later confirmed by a consultant anaesthetist." |
| Why 80:20 first then 90:10? Only 10 test samples? | We describe it between line 254 to 271. The initial 80:20 random split is carried out on the entire available dataset to create the two random main subsets: train (952 samples) and test (238 samples). The 80/20 split is a standard practice in machine learning development to balance between training and testing data.  Subsequently, a 90:10 random split of the training set (952 samples) is used during the 10-fold cross-validation grid search which is the method we employ to train the model and identify the best combination of its parameters. In every iteration 857 samples are used to train the model and 95 are used as the validation set. With this process, also established in machine learning development, we extract the best model parameters by measuring the model’s performance in the validation set during the grid search. This approach allows for granular understanding of the model's behaviour and performance during training.  Once the final model weights and parameters are finalised, testing the model takes place in 10 runs with a 90% random set from the test set (238 samples). This amounts to 214 test samples for each test run.  This method provides a comprehensive evaluation of the model’s testing behaviour, ensuring that the performance metrics are not skewed by a single test run, offering a reliable assessment of the model's capabilities. |

| **Results** |  |
| --- | --- |
| **Reviewer’s comments** | **Author’s comments** |
| In comorbidities (table 1), include renal dysfunction if you have those numbers available because it is a commonly used parameter in many risk scores (Lee’s RCRI). | In Table 1, we have included the parameters Urea, Creatinine, and eGFR. These are essential indicators of renal function and can be used to assess renal dysfunction |
| 69% of patients male. Why do you think such a big difference between males and females? | The higher prevalence of male patients, accounting for 69% of the total, can be attributed to the epidemiology of the surgeries performed at our hospital. Urological conditions often have a higher incidence in males (73% of bladder cancers in the UK are in males, 100% of prostate cancers), which naturally leads to a greater number of male patients undergoing these procedures. Similarly, in the UK, 69% of head and neck cancers are in men. Even colorectal carcinoma, which is more balanced, is more likely to occur in men in the UK (56% male vs 44% female) (Cancer Research UK figures). |
| Mortality rates-Is this in keeping with other studies rates? As UCLH is a first-world tertiary hospital so care may be different to other hospitals which affects generalisability of findings (I know that this is mentioned as a limitation but maybe just comment on whether the mortality rate is similar to other hospitals?) | Yes, as described in the papers cited from line 473-476, the mortality rate at UCLH is comparable with that reported in the literature (our 28-day mortality 2% ; Moonesinghe et al [5]: All patient mortality 1.1%, General surgical mortality 2.4%; EUSOS [43]: UK Mortality 3.6%, Whole study 4%). |
| Table 2: some of the AUC values are very high? Makes me question the inputs. I think the model might need some re-working, especially overlapping inputs. | As stated between lines 254–271, we confirm that no data leakage occurred between the training and testing sets, as they were strictly partitioned at the outset using an 80:20 random split. The test set remained completely independent and was not used at any stage of model training, hyperparameter tuning, or cross-validation. Furthermore, each test run utilized a randomly selected 90% subset of the test set, ensuring that model performance was evaluated exclusively on unseen data.  We acknowledge the reviewers' concern regarding the high AUC values and have carefully examined the potential factors contributing to these results. Importantly, the MOSR algorithm does not exhibit uniformly superior performance across all evaluation metrics. While it consistently achieves higher AUC and F1 scores compared to other models, the XGB (Extreme Gradient Boosting) algorithm demonstrates better accuracy, indicating that no single model dominates across all performance measures.  Our 10-fold cross-validation grid search was conducted strictly within the training set (952 samples), with a 90:10 split in each fold (857 for training, 95 for validation), preventing any potential contamination from the test set. This methodological approach ensures that model tuning and selection were performed without access to the final evaluation data.  Our primary goal was to assess the impact of integrating CRF data alongside the Clinical Dataset to enhance predictive performance. The observed improvements with MOSR remain within a reasonable range, reinforcing the robustness and validity of our approach rather than suggesting an inflated performance due to data leakage. To further substantiate these findings, we plan to validate the model on an independent external dataset, which will provide additional insight into its generalizability and potential overfitting. Furthermore, a key advantage of MOSR is its ability to produce interpretable formulae that can be externally validated, eliminating any risk of reliance on overlapping inputs. |
| SHAP plot: why is work rate at AT and work rate at peak exercise inverse? Surely the higher the work rate at both times, the worse the outcomes? | Thank you for this insightful question regarding the SHAP (SHapley Additive exPlanations) plot and the observed inverse relationship between work rate at anaerobic threshold (AT) and work rate at peak exercise. This observation may initially seem counterintuitive, as one might expect higher work rates at both points to correlate with worse outcomes.  To address this, it is important to consider the physiological context and the specific dataset characteristics. The work rate at AT and peak exercise can reflect different aspects of an individual's cardiovascular and muscular efficiency. Specifically, it is worth considering what AT represents; which is the point in ramped exercise at which the products of anaerobic metabolism overcome intrinsic buffering mechanisms to lower the blood pH and hence cause a relative increase in ventilation (hyperventilation in relation to oxygen usage) to offset this effect. The lower the work rate at which this occurs, the earlier limiting anaerobic metabolism predominates. This might be expected to reflect a similar pattern in terms of response to increases in postoperative metabolic rate and reflects the observed finding in the SHAP analysis that both low AT and low work rate at AT are associated with increased morbidity. The AT therefore , although often associated with peak VO2 (i.e. high peak oxygen consumption is associated with higher AT), is a separate phenomenon and identifies linked but ultimately independent physiological processes than peak VO2.  It would follow then that a lower WR at peak VO2 should predict morbidity, which it does not seem to do in this analysis. We believe this is an indexing phenomenon where oxygen consumption per/kg body weight becomes the explanatory variable. In the reported SHAP analysis, peak oxygen consumption ***indexed to body weight*** has the predicted (and widely reported) relationship of low peak/maximal oxygen consumption (VO2) predicting morbidity and high peak VO2 being associated with reduced morbidity. However, in the SHAP analysis, BMI has a strong impact on model performance, with low BMI being less predictive of morbidity. It is possible that WR at VO2 peak is subject to a paradoxical reversal since lighter people are likely to produce lower absolute work rates. If indexed to body weight, we would expect the relationship to normalize and for work rate at peak VO2 to relate to morbidity in the same way as with AT. These data are not routinely reported in CPET so were not analyzed. |
| SHAP plot: how was operation severity determined? | The operation severity in the SHAP plot was determined using the SORT (Surgical Outcome Risk Tool) score related operation severity value present in the database. The developers of this tool describe their methodology as such: *‘A comparison of the surgical severity for all orthopaedic and gastrointestinal surgical procedures (the two most common surgical specialty groups in the study sample) was made to assess similarities between the AXA and British United Provident Association (BUPA) coding schedules, as the BUPA schedule has been used in previous studies. Clinical judgement was used for procedures falling under more than one surgical type, and where there was no listing for a procedure in the AXA schedule. Two clinical reviewers reached agreement on how to classify such procedures.”* |
| Figure 2F: why when clinical dataset combined with CRF dataset did it degrade model outcomes? | We believe the observed effect results from a significant increase in the number of variables in the combined dataset without a corresponding rise in the number of patients. This imbalance may have introduced confounding factors that affected the machine learning algorithms, ultimately leading to a decline in performance. We also contend that this reflects the difference between disease labels and measured physiology. For example, two age matched individuals with hypertension from an epidemiological point of view will have an increased risk of perioperative cardiovascular complications. However, the label [hypertension] tells us very little about the severity of hypertension in the individual with consequent impact on physiology, hence the impact on model performance will be to degrade it. On the other hand, a time series CPET will reflect the physiological impact that the hypertension has had on cardiovascular performance in that individual, hence improving model performance. That is why we conclude “Additionally, leveraging data directly from the CPET time-series appears to enhance model performance, underscoring the value of examining elder patient physiology and adaptability to acute stress.” |
| Figure 3A: was cleaning done on the dataset as outliers are clearly visible and could skew data. Also, this would better be shown as a box-whisker plot at each time interval. | Each individual plot in figure 3A represents the oxygen uptake for a single patient during the course of a single exercise test plotted in a standardized way as normally reported. Each individual plot is subject to smoothing at time of analysis (middle 5 of 7, which is the standard as per POETTS guidance). We agree that a box-whisker plot would also show the relationships between POMS 0-1 and POMS >2 at each time point, but plotted the figure in this way since visually we feel it conveys the variability in the raw data better. Furthermore, we do not want to convey the message that one feature (in this case VO2) is entirely responsible for model performance, but rather want to illustrate the methods by which we approached the complexity of CPET derived time series data with one of the multiple data points available as an example. With regards to outliers, we accept there is one patient who clearly has a significantly higher performance than the rest of the population, but overall does not affect model performance. None of the curves deviate significantly enough from the distribution to be statistically classified as outliers, so no outlier removal was performed. Only patients with incomplete curves were excluded from the dataset to avoid introducing alterations through data imputation techniques. |
| Figure 3B: why was this smoothing done? Makes the picture prettier but might lose important nuances in dataset. | Smoothing is standard practice for raw CPET data (see above comment and POETTS guidance: <https://ipoetts.org/Clinical-Guidelines>) It was not applied for aesthetic purposes but to manage the noise generated by raw indirect metabolic cart data while preserving the curve's essential shape and informative content. Extending this process also enabled us to obtain a representative dataset with fewer data points, helping ML algorithms focus on the most relevant aspects of the data rather than being influenced by machine-generated noise. |

| **Discussion** |  |
| --- | --- |
| **Reviewer’s comments** | **Author’s comments** |
| Cardiorespiratory fitness written out in full. Use CRF throughout. | The text has been corrected to use the abbreviation CRF for cardiorespiratory fitness throughout. |
| Going forward, we want to use these models to counsel patients regarding their risk. A couple of questions/comments/potential research opportunities.   1) Can you perform surgery specific models vs general models to adequately counsel patients for individual surgeries 2) Consider incorporating biomarkers such as proBNP into future models | 1) Yes, this can be done by expanding the patient pool for each specific surgery. With sufficient data, surgery-specific models can be developed to provide more tailored risk assessments.  2) Incorporating biomarkers such as proBNP is a viable approach. The model could quantify its impact and reveal how much it enhances predictive performance as well as influence on named outcomes such as the development of Myocardial Injury after non cardiac surgery. This is the basis of work currently under development. |

PDIG-D-24-00447

Assessing perioperative risks in a mixed elderly surgical population: a multi-objective symbolic regression approach to cardiorespiratory fitness derived from cardiopulmonary exercise testing

PLOS Digital Health

Dear Dr. Arina,

Thank you for submitting your manuscript to PLOS Digital Health. After careful consideration, we feel that it has merit but does not fully meet PLOS Digital Health's publication criteria as it currently stands. Therefore, we invite you to submit a revised version of the manuscript that addresses the points raised during the review process.

Please submit your revised manuscript within 60 days Mar 14 2025 11:59PM. If you will need more time than this to complete your revisions, please reply to this message or contact the journal office at [digitalhealth@plos.org](mailto:digitalhealth@plos.org). When you're ready to submit your revision, log on to [https://www.editorialmanager.com/pdig/](https://eur01.safelinks.protection.outlook.com/?url=https%3A%2F%2Fwww.editorialmanager.com%2Fpdig%2F&data=05%7C02%7Cp.arina%40ucl.ac.uk%7C7c48ea416755456e595708dd348bd2ec%7C1faf88fea9984c5b93c9210a11d9a5c2%7C0%7C0%7C638724497378978650%7CUnknown%7CTWFpbGZsb3d8eyJFbXB0eU1hcGkiOnRydWUsIlYiOiIwLjAuMDAwMCIsIlAiOiJXaW4zMiIsIkFOIjoiTWFpbCIsIldUIjoyfQ%3D%3D%7C0%7C%7C%7C&sdata=IqQ8q5xYxINTBToWCqkwS9TAgK2FNeHxHLnROe3dqIo%3D&reserved=0) and select the 'Submissions Needing Revision' folder to locate your manuscript file.

Please include the following items when submitting your revised manuscript:

* A rebuttal letter that responds to each point raised by the editor and reviewer(s). You should upload this letter as a separate file labeled 'Response to Reviewers'. This file does not need to include responses to any formatting updates and technical items listed in the 'Journal Requirements' section below.

* A marked-up copy of your manuscript that highlights changes made to the original version. You should upload this as a separate file labeled 'Revised Manuscript with Track Changes'.

* An unmarked version of your revised paper without tracked changes. You should upload this as a separate file labeled 'Manuscript'.

If you would like to make changes to your financial disclosure, competing interests statement, or data availability statement, please make these updates within the submission form at the time of resubmission. Guidelines for resubmitting your figure files are available below the reviewer comments at the end of this letter.

We look forward to receiving your revised manuscript.

Kind regards,

Dhiya Al-Jumeily OBE, PhD

Section Editor

PLOS Digital Health

Leo Anthony Celi

Editor-in-Chief

PLOS Digital Health

orcid.org/0000-0001-6712-6626

**Journal Requirements:**

1. Please provide separate figure files in .tif or .eps format.

For more information about figure files please see our guidelines:
[https://journals.plos.org/digitalhealth/s/figures](https://eur01.safelinks.protection.outlook.com/?url=https%3A%2F%2Fjournals.plos.org%2Fdigitalhealth%2Fs%2Ffigures&data=05%7C02%7Cp.arina%40ucl.ac.uk%7C7c48ea416755456e595708dd348bd2ec%7C1faf88fea9984c5b93c9210a11d9a5c2%7C0%7C0%7C638724497378995242%7CUnknown%7CTWFpbGZsb3d8eyJFbXB0eU1hcGkiOnRydWUsIlYiOiIwLjAuMDAwMCIsIlAiOiJXaW4zMiIsIkFOIjoiTWFpbCIsIldUIjoyfQ%3D%3D%7C0%7C%7C%7C&sdata=FAu9WKYkeeP%2BHwxoAXqYf6nf30EjArRSnIuVRysPlYU%3D&reserved=0) 
[https://journals.plos.org/digitalhealth/s/figures#loc-file-requirements](https://eur01.safelinks.protection.outlook.com/?url=https%3A%2F%2Fjournals.plos.org%2Fdigitalhealth%2Fs%2Ffigures%23loc-file-requirements&data=05%7C02%7Cp.arina%40ucl.ac.uk%7C7c48ea416755456e595708dd348bd2ec%7C1faf88fea9984c5b93c9210a11d9a5c2%7C0%7C0%7C638724497379011909%7CUnknown%7CTWFpbGZsb3d8eyJFbXB0eU1hcGkiOnRydWUsIlYiOiIwLjAuMDAwMCIsIlAiOiJXaW4zMiIsIkFOIjoiTWFpbCIsIldUIjoyfQ%3D%3D%7C0%7C%7C%7C&sdata=FT8IQsUv7x6SG4y8nu0Z6ORdSVznVL5y7SgpdPUbrqs%3D&reserved=0)

2. Please provide an Author Summary. This should appear in your manuscript between the Abstract (if applicable) and the Introduction, and should be 150–200 words long. The aim should be to make your findings accessible to a wide audience that includes both scientists and non-scientists. Sample summaries can be found on our website under Submission Guidelines:
[https://journals.plos.org/globalpublichealth/s/submission-guidelines#loc-parts-of-a-submission](https://eur01.safelinks.protection.outlook.com/?url=https%3A%2F%2Fjournals.plos.org%2Fglobalpublichealth%2Fs%2Fsubmission-guidelines%23loc-parts-of-a-submission&data=05%7C02%7Cp.arina%40ucl.ac.uk%7C7c48ea416755456e595708dd348bd2ec%7C1faf88fea9984c5b93c9210a11d9a5c2%7C0%7C0%7C638724497379031001%7CUnknown%7CTWFpbGZsb3d8eyJFbXB0eU1hcGkiOnRydWUsIlYiOiIwLjAuMDAwMCIsIlAiOiJXaW4zMiIsIkFOIjoiTWFpbCIsIldUIjoyfQ%3D%3D%7C0%7C%7C%7C&sdata=A7LI39NEq6MoVCIz9nqReRgESrTwVPhRkEDsG6n0%2Fig%3D&reserved=0).

**Additional Editor Comments (if provided):**

Revise please considering the reviewers comments. Specific attention should be paid to the writing of the paper and the citations. Please cite statements where relevant and revise citations and references.

[Note: HTML markup is below. Please do not edit.]

**Reviewers' Comments:**

Reviewer #1: Manuscript #: PDIG-D-24-00447
Title: Assessing perioperative risks in a mixed elderly surgical population: a multi-objective symbolic regression approach to cardiorespiratory fitness derived from cardiopulmonary exercise testing
By Pietro Arina et al.

The authors explored the potential of integrating cardiorespiratory fitness (CRF) data from cardiopulmonary exercise testing (CPET) into machine learning (ML) models to improve the accuracy of preoperative risk assessment for predicting postoperative morbidity in elderly patients undergoing major elective surgery. The study found that incorporating CRF data significantly enhanced the performance of all models, with the Multi-Objective Symbolic Regression (MOSR) model, a novel algorithm based on genetic programming, consistently outperforming traditional risk scores and other ML approaches. The authors also examined the impact of using CPET time-series data directly, demonstrating a further improvement in model performance over models based on manually extracted CPET features. The findings suggest that incorporating CPET-derived CRF data into ML models has the potential to revolutionize perioperative risk prediction in elderly surgical patients, potentially leading to more targeted interventions and improved patient outcomes.

The use symbolic regression approach to model the CRF data is a major strength. The manuscript, however, is written in a rush, with many glaring errors, which renders the paper quality extremely poor. The overall findings are interesting but can be further improved. The model discovered by symbolic regression needs some elaborations/explanations for the variables involved, perhaps some links to the SHAP features. This would be more interesting than simple prediction. Overall, figure legends are way too small to read. Figure quality and some explanations need improvement. Other specific comments are as follows:

1. Of 2,145 patients assessed, 1,190 were included in the tabular database, and 585 with full CPET recordings for time-series analysis, but in Supplementary Figure 1, by Excluded n=1005, there are only 1140, not 1190 patients. The patient number does not match.

Thank you, typos corrected, N=995.
2. What are the purposes to have Major and Minor POMS morbidity classification?

Line 208- The purpose of having Major and Minor POMS morbidity classifications is to account for the varying clinical significance of different postoperative morbidities. The POMS classification broadly accounts for any morbidity that has the potential to keep a patient in hospital. Not all POMS events carry the same impact—there is a clear distinction between, for example, respiratory impairment and the presence of a surgical drain. Based on existing literature, we categorized POMS into minor (mild factors) and major (severe factors) to provide an additional layer of patient stratification, allowing for a more nuanced assessment of postoperative risk and outcomes.
3. Which software is used for MOSR analysis? It would be good to make the code public available, and even better if you could make the data available.

Added to line 278: The ML library for MOSR is open source and available at <https://github.com/davideferrari92/multiobjective_symbolic_regression>

Data cannot be made public because the conflict with the current ethical approval for this study and the difficulty in properly anonymizing this type of data.
4. In the Abstract, it says “A model was also developed for the same task using data directly extracted from the CPET time-series.”. Which model do you refer to? And what are the conclusions of this model?

Line 84 - This refers to the MOSR model built using resampled CPET time series directly, rather than relying on a set of manually pre-processed features extracted to highlight specific clinically relevant characteristics, such as the curve’s peak value and the slope of the recovery phase.
5. The reference in the paper are terribly messed up, some places using authors, year e.g. the Lee et al., 2022; J. Rose et al., 2015; Lee et al., 2022)) , but other place using number, fpr example, [25]. There are many problems: 1. Repeated reference: “Lee et al., 2022; J. Rose et al., 2015; Lee et al., 2022))”; 2. “DenckerTjeertes et al., 2021)2016)” unsure what it is; 3. Ferrari, Guidetti, Wang, et al., 2022b2022), et c etc …

The references have been corrected, addressing the previous issues related to Mendeley. They are now formatted correctly using the PLOS ONE citation style, ensuring consistency and adherence to the journal's guidelines.
6. Not complete sentence, “dherence to Caldicott principles ensured data confidentiality and proper collection methods”, no period at the end of sentence.

Line 158 Corrected
7. Instead of simply listing the statistics used, “statistical tests such as Student’s t-test, Mann-Whitney U test, Chi-Squared test, and Fisher’s Exact Test were utilized.”, I suggest to explicitly indicate each test when used.
Corrected, added the clarifications to lines 338, 342

8. Why testing runs on a random 90% sample (214) of the testing set, but not the whole testing data?

We reported it between line 254 to 271.
The initial 80:20 random split is carried out on the entire available dataset to create the two random main subsets: train (952 samples) and test (238 samples). The 80/20 split is a standard practice in machine learning development to balance between training and testing data.

Subsequently, a 90:10 random split of the training set (952 samples) is used during the 10-fold cross-validation grid search which is the method we employ to train the model and identify the best combination of its parameters. In every iteration 857 samples are used to train the model and 95 are used as the validation set. With this process, also established in machine learning development, we extract the best model parameters by measuring the model’s performance in the validation set during the grid search. This approach allows for granular understanding of the model's behaviour and performance during training.

Once the final model weights and parameters are finalised, testing the model takes place in 10 runs with a 90% random set from the test set (238 samples). This amounts to 214 test samples for each test run.

This method provides a comprehensive evaluation of the model’s testing behaviour, ensuring that the performance metrics are not skewed by a single test run, offering a reliable assessment of the model's capabilities.

9. “e evolved a population of 300 individual models for 500 generations.”, why 300 and 500 used?

The choice of evolving a population of 300 individual models for 500 generations was based on experimentally validated optimum performance. This decision was informed by the authors' previous experiences and findings, as demonstrated in the cited papers, papers in bibliography n19 and 20. These numbers were found to provide a balance between computational efficiency and the ability to achieve significant evolutionary adaptation, ensuring robust model performance.

10. Why the model performance with more information by using both CRF and clinical data gets worse than using either data alone?

We believe the observed effect results from a significant increase in the number of variables in the combined dataset without a corresponding rise in the number of patients. This imbalance may have introduced confounding factors that affected the machine learning algorithms, ultimately leading to a decline in performance. We believe the observed effect results from a significant increase in the number of variables in the combined dataset without a corresponding rise in the number of patients. This imbalance may have introduced confounding factors that affected the machine learning algorithms, ultimately leading to a decline in performance. We also contend that this reflects the difference between disease labels and measured physiology. For example, two age matched individuals with hypertension from an epidemiological point of view will have an increased risk of perioperative cardiovascular complications. However, the label [hypertension] tells us very little about the severity of hypertension in the individual with consequent impact on physiology, hence the impact on model performance will be to degrade it. On the other hand, a time series CPET will reflect the physiological impact that the hypertension has had on cardiovascular performance in that individual, hence improving model performance. That is why we conclude “Additionally, leveraging data directly from the CPET time-series appears to enhance model performance, underscoring the value of examining elder patient physiology and adaptability to acute stress.”

11. Fig.2c, “Cardiopulmonary Exercise Testing (CPET) (red),”, there is no red curve.

The CPET curve is the orange (VO2/Kg AT), typo corrected

Reviewer #2: Assessing perioperative risks in a mixed elderly surgical population

Introduction
The first sentence is a little awkward, suggest revising.
“Accurate preoperative risk prediction supports patients in making informed decisions and guides clinical decision-making.” Is risk prediction for the patient or clinician?
For the benefit of both: patients will be supported in shared decision making with data that is personalized to them, clinicians will be supported in clinical decision making with risk data that may inform the appropriateness of potential clinical treatment options e.g. selection of appropriate surgical/non surgical treatment options

Try to limit abbreviations; a number of times I had to go back to find what some meant
Thank you, we have reduced the number of abbreviations in the manuscript

Methods
There is no mention of inclusion/exclusion criteria in text. How were the patients selected? Particularly those that were included.

The inclusion/exclusion criteria are described in the text (lines 163 to 168) and supplementary figure 1: broadly we included all people aged 18 of over routinely referred for CPET as part of their preoperative assessment. People who were unable to perform CPET due to medical exclusions per iPOETTS guidance were excluded (see main text…) as were those with missing outcomes data (see CONSORT diagram). We have modified the Population and Selection Criteria section slightly to clarify further.

How was the POMS administered? In hospital/home? Free of people/distractions or was it supervised by a researcher?

POMS was prospectively recorded by trained researchers, including clinicians and nurses, in a controlled hospital environment to ensure consistency and accuracy. This has, for many years been a core data point collected by our SOURCE (<http://www.uclsource.com>) team employed by our research department. Line 213 has been updated to reflect the collection methodology.

Table 1 – “CPET values” – these just appear to be resting physiological variables independent of CPET.

Corrected

“VE/V̇CO2 (ml/min)” – VE/VCO2 doesn’t have a unit as far as I am aware as it is a ratio.
Corrected

Results
“A modest correlation existed between advancing age and an increased
number of POMS-positive domains at day 3, as indicated by the POMS score (Figure-1C)” It would be helpful here and else where to report the correlation (with 95% CI).
Corrected in line 336 and line 340, reported the correlation coefficient.

Discussion
It would be helpful to explain some of your complex statistical results, in a more lay format. For example, what does an improvement of 49% for PPV mean? How many more patients are accurately captured or how many are still missed?

We have significantly rewritten the discussion to incorporate this feedback while keeping the original meaning intact.

“The best model, utilizing the MOSR algorithm and incorporating CRF data, demonstrated an average increase of 27% in AUC and a 22% improvement in F1-score” What does this mean for a clinician, in practical terms?

Please see above

The term “fitness response” does not make sense…do you mean CRF or exercise response

This has now been removed

Reviewer #3: I am concerned about the accuracy of the model. The AUC values are very high which makes me questions the inputs. Are there too many overlapping inputs? Consider splitting some of the dataset in the inputs to see which one is degrading the outcomes. The SHAP plots and some of the figures need revision.

Please see response in the table above

[NOTE: If reviewer comments were submitted as an attachment file, they will be attached to this email and accessible via the submission site. Please log into your account, locate the manuscript record, and check for the action link "View Attachments". If this link does not appear, there are no attachment files.]

**Figure resubmission:**

While revising your submission, please upload your figure files to the Preflight Analysis and Conversion Engine (PACE) digital diagnostic tool, [https://pacev2.apexcovantage.com/](https://eur01.safelinks.protection.outlook.com/?url=https%3A%2F%2Fpacev2.apexcovantage.com%2F&data=05%7C02%7Cp.arina%40ucl.ac.uk%7C7c48ea416755456e595708dd348bd2ec%7C1faf88fea9984c5b93c9210a11d9a5c2%7C0%7C0%7C638724497379047094%7CUnknown%7CTWFpbGZsb3d8eyJFbXB0eU1hcGkiOnRydWUsIlYiOiIwLjAuMDAwMCIsIlAiOiJXaW4zMiIsIkFOIjoiTWFpbCIsIldUIjoyfQ%3D%3D%7C0%7C%7C%7C&sdata=VJNn%2BzTIOEt0wykFurYah93dPL%2FfBqXdYU%2Bd8RAY3F0%3D&reserved=0). PACE helps ensure that figures meet PLOS requirements. To use PACE, you must first register as a user. Registration is free. Then, login and navigate to the UPLOAD tab, where you will find detailed instructions on how to use the tool. If you encounter any issues or have any questions when using PACE, please email PLOS at [figures@plos.org](mailto:figures@plos.org). Please note that Supporting Information files do not need this step. If there are other versions of figure files still present in your submission file inventory at resubmission, please replace them with the PACE-processed versions.

**Reproducibility:**

To enhance the reproducibility of your results, we recommend that authors of applicable studies deposit laboratory protocols in protocols.io, where a protocol can be assigned its own identifier (DOI) such that it can be cited independently in the future. Additionally, PLOS ONE offers an option to publish peer-reviewed clinical study protocols. Read more information on sharing protocols at [https://plos.org/protocols?utm_medium=editorial-email&utm_source=authorletters&utm_campaign=protocols](https://eur01.safelinks.protection.outlook.com/?url=https%3A%2F%2Fplos.org%2Fprotocols%3Futm_medium%3Deditorial-email%26utm_source%3Dauthorletters%26utm_campaign%3Dprotocols&data=05%7C02%7Cp.arina%40ucl.ac.uk%7C7c48ea416755456e595708dd348bd2ec%7C1faf88fea9984c5b93c9210a11d9a5c2%7C0%7C0%7C638724497379060911%7CUnknown%7CTWFpbGZsb3d8eyJFbXB0eU1hcGkiOnRydWUsIlYiOiIwLjAuMDAwMCIsIlAiOiJXaW4zMiIsIkFOIjoiTWFpbCIsIldUIjoyfQ%3D%3D%7C0%7C%7C%7C&sdata=Wvur55NDm0672ud4Cp1IqNsp4ZXndJytofDzAzkAKMI%3D&reserved=0)
